# Response to: Comment on “Establishing a Porcine Model of Small for Size Syndrome following Liver Resection”

**DOI:** 10.1155/2018/7565408

**Published:** 2018-08-19

**Authors:** Mohammad Golriz, Elias Khajeh, Omid Ghamarnejad, Arianeb Mehrabi

**Affiliations:** Department of General, Visceral, and Transplantation Surgery, University of Heidelberg, Heidelberg, Germany

With great interest we read the most recent letter of Athanasiou et al. [1], this time about our publication [2]. The mentioned issues can be categorized in the following subjects:

*Transhepatic Flow (THF) Variations and Small for Size and Flow Syndrome (SFSF)*. The important role of THF variations in SFSF has been clarified by our group previously [3]. However, the mechanism and definition of SFSF should not be confused with one another. Variations in THF cause SFSF and can be considered a prerequisite factor for SFSF [4], but THF variations are not necessary for confirmation of the diagnosis. Moreover, we have previously showed the THF variations following extended liver resection [5, 6]. SFSF is diagnosed based on the clinical, laboratory, and, if possible, histopathological findings [7]. Hemodynamic variations during and after liver resection are measured to correlate these values with the diagnosis and to define prognostic cut-off values. Measuring hemodynamic variations could add extra information to our study but was not necessary to confirm the diagnosis.

*Remnant Liver Volume (RLV) and SFSF*. SFSF and posthepatectomy liver failure (PHLF) are often mistaken as the same, and this is shown in the comments made by Athanasiou. This mistake is common because SFSF and PHLF are usually overlapping [8]. However, SFSF is a clinical syndrome after liver resection which can lead to irreversible PHLF but can also be prevented from ending in that. SFSF causes PHLF because of small RLV and increased portal vein flow per 100 gr remnant liver. An optimal animal model of SFSF for evaluating the preventive, diagnostic, and therapeutic procedures has to mirror the deterioration in liver function and have the capacity to be compensated or reversed. In the clinical setting, no surgeon will resect that much liver to make PHLF and death inevitable [9, 10]. In other words, an SFSF model should be reversible and may be rescued by intervention. However, most of the animals in SFSF model die from PHLF if no intervention is received. This can be reached in porcine model through a trisectionectomy [11–15]. Moreover, the resection cut-off level depends on the size of segments 1, 6, and 7. If these segments are large, it is sometimes necessary to resect a further 5% to achieve the cut-off level [16, 17]. Resection that causes early death without the possibility for potential compensation (irreversible) is not an optimal SFSF model. To establish and understand a proper animal model, enough experience with the anatomy and physiology of the animal is required, especially in the respective field [18–24].

*Triggers of Liver Regeneration*. Triggers of liver regeneration have to be differentiated from liver regeneration itself. It is true that hypoxia may trigger liver regeneration [25]. However, constant hypoxia causes liver failure. Hypertrophy after liver resection is not explained by hypoxia; it is triggered by hypoxia. Moreover, the arterial buffer response cannot be reversed [26, 27].

*Summary*. SFSF following extended liver resection is a complex process that is often mistaken with liver failure after partial liver transplantation or considered as equal to PHLF. However, SFSF is a clinical syndrome after liver resection which can lead to irreversible PHLF but can also be prevented from ending in that. In other word, every SFSF is a reversible PHLF which can end in irreversible PHLF.
